# Analysis of urinary C–C motif chemokine ligand 14 (CCL14) and first-generation urinary biomarkers for predicting renal recovery from acute kidney injury: a prospective exploratory study

**DOI:** 10.1186/s40560-023-00659-2

**Published:** 2023-03-20

**Authors:** Ben-Shu Qian, Hui-Miao Jia, Yi-Bing Weng, Xin-Cheng Li, Chao-Dong Chen, Fang-Xing Guo, Yu-Zhen Han, Li-Feng Huang, Yue Zheng, Wen-Xiong Li

**Affiliations:** 1 Department of Surgical Intensive Critical Unit, Beijing Chao-Yang Hospital, Capital Medical University, 8 Gongren Tiyuchang Nanlu, Chaoyang District, Beijing, 100020 China; 2 Department of Emergent Intensive Critical Unit, Beijing Lu-He Hospital, Capital Medical University, Beijing, 101100 China

**Keywords:** TIMP-2, IGFBP7, CCL14, NGAL, Acute kidney injury, Renal recovery, Prognosis

## Abstract

**Background:**

Acute kidney injury (AKI) is a frequent syndrome in the intensive care unit (ICU). AKI patients with kidney function recovery have better short-term and long-term prognoses compared with those with non-recovery. Numerous studies focus on biomarkers to distinguish them. To better understand the predictive performance of urinary biomarkers of renal recovery in patients with AKI, we evaluated C–C motif chemokine ligand 14 (CCL14) and two first-generation biomarkers (cell cycle arrest biomarkers and neutrophil gelatinase-associated lipocalin) in two ICU settings.

**Methods:**

We performed a prospective study to analyze urinary biomarkers for predicting renal recovery from AKI. Patients who developed AKI after ICU admission were enrolled and urinary biomarkers including tissue inhibitor of metalloproteinase-2 (TIMP-2), insulin-like growth factor-binding protein 7 (IGFBP7), CCL14, and neutrophil gelatinase-associated lipocalin (NGAL) were detected on the day of AKI diagnosis. The primary endpoint was non-recovery from AKI within 7 days. The individual discriminative ability of CCL14, [TIMP-2] × [IGFBP7] and NGAL to predict renal non-recovery were evaluated by the area under receiver operating characteristics curve (AUC).

**Results:**

Of 164 AKI patients, 64 (39.0%) failed to recover from AKI onset. CCL14 showed a fair prediction ability for renal non-recovery with an AUC of 0.71 (95% CI 0.63–0.77, *p* < 0.001). [TIMP-2] × [IGFBP7] showed the best prediction for renal non-recovery with an AUC of 0.78 (95% CI 0.71–0.84, *p* < 0.001). However, NGAL had no use in predicting non-recovery with an AUC of 0.53 (95% CI 0.45–0.60, *p* = 0.562). A two-parameter model (non-renal SOFA score and AKI stage) predicted renal non-recovery with an AUC of 0.77 (95% CI 0.77–0.83, *p* = 0.004). When [TIMP-2] × [IGFBP7] was combined with the clinical factors, the AUC was significantly improved to 0.82 (95% CI 0.74–0.87, *p* = 0.049).

**Conclusions:**

Urinary CCL14 and [TIMP-2] × [IGFBP7] were fair predictors of renal non-recovery from AKI. Combing urinary [TIMP-2] × [IGFBP7] with a clinical model consisting of non-renal SOFA score and AKI stage enhanced the predictive power for renal non-recovery. Urinary CCL14 showed no significant advantage in predicting renal non-recovery compared to [TIMP-2] × [IGFBP7].

**Supplementary Information:**

The online version contains supplementary material available at 10.1186/s40560-023-00659-2.

## Background

Acute kidney injury (AKI) is a frequent complication of critical illness, resulting in increased short-term and long-term mortality, significant healthcare costs, and higher risks of chronic kidney disease (CKD) and end-stage renal disease [1–4]. Moreover, many studies indicated that the pattern of AKI recovery affected the prognosis and outcomes [5, 6]. Preventing the non-recovery of renal function should become the therapeutic target of AKI. Therefore, an early biomarker for AKI recovery is needed.

Among AKI biomarkers, urine neutrophil gelatinase-associated lipocalin (NGAL) and the recent combination of urine tissue inhibitor of metalloproteinases-2 and insulin-like growth factor-binding protein 7 ([TIMP-2] × [IGFBP7]) are two first-generation biomarkers that can be used to detect kidney damage and predict AKI before serum creatinine [7–9]. However, only a few studies have assessed the performance of [TIMP-2]*[IGFBP7] as prognosis markers for non-recovery within 48 h or at discharge in patients following cardiac surgery or patients at surgical ICU [10, 11]. Meanwhile, urine C–C motif chemokine ligand 14 (CCL14) was recently reported to have a good even excellent performance in predicting persistent KDIGO stage 3 AKI, with areas under the receiver operating characteristic curves (AUCs) from 0.81 to 0.93 [12–14]. CCL14 is a kind of chemokine released from tubular epithelial cells after injury. CCL14 binds with the C–C chemokine receptors type 1, C–C chemokine receptors type 5, and C–C chemokine receptors type 3 on monocytes and T cells [12, 15]. Renal monocyte recruitment and activation affected the mechanisms of inflammation and fibrosis in kidney tissue damage [16]. Previous work has shown that CCL14 is one of inflammatory markers mediating the risk of progression to end-stage renal disease in diabetes [17]. Hence, CCL14 as an independent predictor of renal recovery from AKI is biologically plausible. However, no study has explored the predictive role of urine CCL14 for renal non-recovery from AKI. Now, we for the first time report an exploratory comparison of urine CCL14 and first-generation urinary biomarkers in predicting non-recovery in critically ill patients with AKI.

## Methods

### Study design and ethics

The study is a prospective exploratory study designed to assess the predictive value of urinary biomarkers for renal non-recovery from AKI. The study conformed to the provisions of the Declaration of Helsinki. Ethical approval was obtained from the Human Ethics Committee of Beijing Chao-yang Hospital, Capital Medical University (ethics number 2018-117). Written informed consent was obtained from patients or their delegates. Study design and manuscript preparation followed the Standards for Reporting Diagnostic Accuracy (STARD) statement [18].

### Participants

The present study was performed in two ICUs at different Chinese tertiary hospitals. We screened patients admitted to the ICUs from October 2020 to May 2022. Patients with new-onset AKI were prospectively and consecutively enrolled. Exclusion criteria included: (1) age < 18 years; (2) established AKI before ICU admission; and (3) failure to obtain adequate urine samples. All enrolled patients adhered to the same management principles as follows: the KDIGO bundle consisting of optimization of volume status, maintenance of perfusion pressure, discontinuation of nephrotoxic drugs and prevention of hyperglycemia [19]; active treatment of primary disease and complications; and the same treatment principles using antibiotics, nutritional metabolism, and organ support. Furthermore, angiotensin-converting enzyme inhibitors/angiotensin receptor blockers and nonsteroidal anti-inflammatory drugs would be discontinued [20].

### Biomarker measurements

Urine samples were collected as soon as AKI was diagnosed. After centrifugation at 3000 rpm for 10 min at 4 °C, the supernatant urine was stored and frozen at − 80 °C until analyzed. The [TIMP-2] × [IGFBP7] was measured using a commercially available NephroCheck Test (Astute Medical, San Diego, CA, USA). NGAL and CCL14 were measured by enzyme-linked immunosorbent assay (ab119600 (NGAL); ab272201 (CCL14), Abcam, UK). The technicians measuring biomarkers were blinded to the clinical data and the physicians responsible were blinded to the results of biomarkers test.

### Outcomes and definitions

The diagnosis of AKI was based on changes in the serum creatinine (SCr) or urine output proposed by the KDIGO guidelines, meeting any of the following: (1) increase in SCr ≥ 0.3 mg/dl (≥ 26.5 µmol/L) within 48 h; (2) increase in SCr to ≥ 1.5 times baseline, which was known or suspected to have occurred within 7 days in the past; (3) urine output < 0.5 mL/kg/h for more than 6 h [21]. Baseline creatinine levels were obtained as follows: if more than 5 values were obtained within 6 months to 7 days prior to admission, the median of all values available was used. Otherwise, the lowest value during the 7 days before admission was used. Assuming a baseline glomerular filtration rate (GFR) of 75 mL/min/1.73 m^2^, the missing baseline creatinine was estimated using the Modification of Diet in Renal Disease formula [22, 23]. CKD was defined as an estimated GFR of less than 60 mL/min/1.73 m^2^ for at least 3 months according to the National Kidney Foundation [24].

The primary endpoint was a failure to recover from AKI within 7 days. We defined renal recovery as the lack of any stage of AKI according to either SCr or urine output criteria. For example, patients with AKI stage 2 had to have a decrease in SCr to below 150% of baseline and be absent in the phase of oliguria (urine output < 0.5 mL/kg/h) for more than 6 h. Patients needing kidney replacement therapy (KRT) on day 7 and those who died after AKI within 7 days were considered renal non-recovery, as renal reversal without survival is rare [5]. For patients diagnosed with both SCr and urine output criteria, the AKI stage was recorded as the more severe one.

The secondary endpoints were the initiation of KRT in the ICU stay, hospital mortality, and 30-day mortality. The KRT was initiated if patients met at least one of the indications (Additional file 1: Table S1) [25].

### Sample size calculation

The formula calculating the sample size for a cohort study was used in this study:

$$n=\frac{{\left({Z}_{\alpha }\sqrt{2\overline{pq} }+{Z}_{\beta }\sqrt{{p}_{0}{q}_{0}+{p}_{1}{q}_{1}}\right)}^{2}}{{\left({p}_{1}-{p}_{0}\right)}^{2}},$$

where *Z* was a statistical value, *p*_1_ and *p*_0_ represented the expected incidence of the exposure group and the non-exposure group, respectively, *q*_0_ = 1 − *p*_0_, *q*_1_ = 1 − *p*_1_, ‾*p* was the average of the two incidences, *q* = 1 − *p*, α = 0.05 and the power (1 − β) was 90%.

Based on our pretest results, the incidence of renal non-recovery was 0.61 for the exposed group (urine CCL14 levels above the threshold) and 0.23 for the non-exposed group (urine CCL14 levels above the threshold). According to the formula mentioned above, the sample size calculated was 68. The same formula was used to calculate sample sizes for [TIMP-2] × [IGFBP7] and NGAL, which were 36 and 154, respectively.

Selecting the largest one of the three, the sample size for this study was 154. Considering the loss rate to follow-up (about 5%), the estimated total sample size was 154 + (154 × 5%) = 162.

### Data collection

The ICU critical care platform in the hospital assisted in prospective collection of clinical data, including patient demographics, prior health history, diagnosis, comorbidities, mechanical ventilation, and use of vasopressors. Patient severity was estimated by Acute Physiology and Chronic Health Evaluation (APACHE II) and Sequential Organ Failure Assessment (SOFA) scores on the day of AKI diagnosis (day 0). Serum creatinine was detected and recorded at ICU admission and every 12 h thereafter until day 7 after AKI. Urine output was measured hourly from the catheter in the ICU period and recorded in the ICU critical care platform. Moreover, death during hospital stay and 30 days after AKI establishment were followed up.

### Statistical analysis

SPSS Statistics 24 and MedCalc software were performed for statistical analysis. Categorical variables were described as percentiles, and continuous variables were described as mean ± standard deviation (SD) or median (Q1, Q3). Continuous data between the two groups (recovery group and non-recovery group) were compared using *t* tests or Mann–Whitney *U* tests, and categorical variables were compared using Chi-square tests or Fisher’s exact tests. We assessed correlations using Spearman’s rank correlation coefficients. For all analyses, a two-sided *p* < 0.05 was considered statistically significant.

The predictive discrimination of each marker and related model was assessed using the area under the receiver-operating characteristic (ROC) curve (AUC). The following values were utilized to describe the AUC: 0.90–1.0, excellent; 0.80–0.89, good; 0.70–0.79, fair; 0.60–0.69, poor; and 0.50–0.59, useless. We used the Youden index to determine the optimal cutoff for calculations of specificity, and sensitivity. Confidence intervals for the AUCs and pairwise comparisons of AUCs were calculated by Delong’s method.

The associations of clinical variables on day 0 with renal non-recovery were evaluated by multivariate logistic regression analysis using a stepwise forward-selection procedure. According to the previous review, clinical parameters associated with renal non-recovery such as age, CKD, comorbidity, illness severity, medical admission, and severity of AKI were included in univariate analysis (*t* tests or Chi-square tests) [7]. Clinical parameters with *p* < 0.15 in the univariate analysis were included in the multivariate logistic regression analysis. Ordinal variables were directly entered into regression analysis. Continuous variables were transformed into categorical variables and regarded as dummy variables. Variables with *p* < 0.05 in the multivariate logistic regression were independent risk factors for renal non-recovery. The sample size of logistic regression analysis met at least 10 events per variable [26]. The contribution of biomarkers to clinical prediction was further investigated by net reclassification improvement (NRI) and integrated discrimination improvement (IDI) methods.

## Results

### Overall patient characteristics and outcomes

**Fig. 1:**
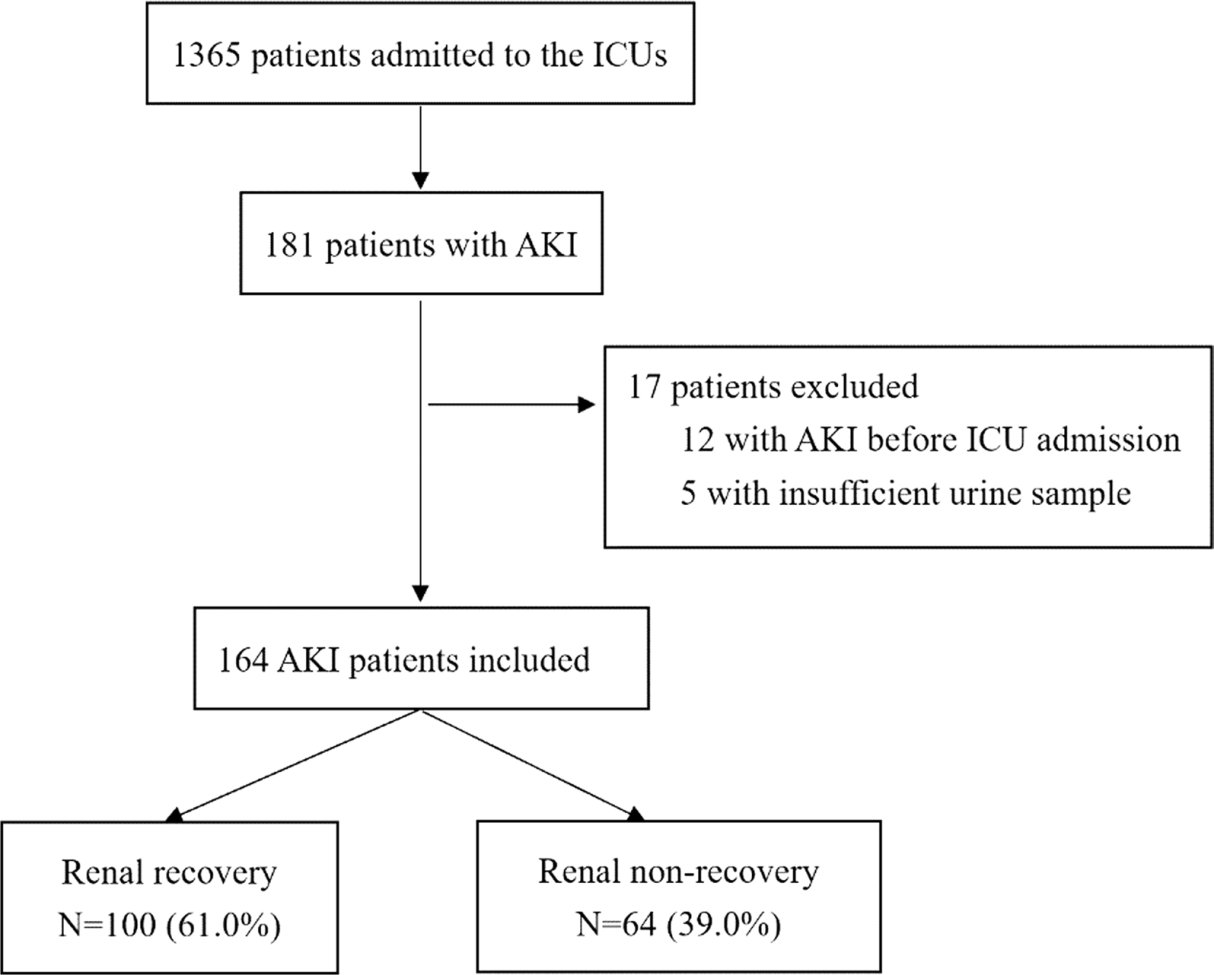
Study flow diagram. *ICU* intensive care unit, *AKI* acute kidney injury

During the study period, 1365 critically ill patients were screened, and 181 (13.3%) of them developed AKI. 164 patients with AKI were ultimately enrolled after excluding 17 ineligible patients. All of the patients were Asian. 100 (61.0%) patients had renal recovery from AKI occurrence within 7 days, and 64 (39.0%) patients encountered renal non-recovery (Fig. 1).

Baseline clinical characteristics and outcomes are shown in Table 1. There were no significant differences in demographic characteristics, comorbidities, and nephrotoxic drug use. However, patients who failed to recover had remarkably higher APACHE II scores and non-renal SOFA scores than those who successfully recovered. Besides, in terms of kidney features, the distribution of serum creatinine, eGFR, urine output, and AKI stage at enrollment showed significant differences between patients with and without renal recovery. In the non-recovery group, a more proportion of patients were postoperative and emergency sources.

**Table 1:** Patient characteristics between patients with and without renal recovery

	Recovery (*n* = 100)	Non-recovery (*n* = 64)	*p value*
Age (years)	60 (46, 72)	56 (43, 66)	0.259
Male gender	65 (65.0)	37 (57.8)	0.410
Body mass index (kg/m^2^)	22.9 (20.6, 25.4)	23.9 (20.4, 27.0)	0.162
APACHE II score	13.0 (11, 17)	17 (12, 22)	0.014
Non-renal SOFA score	4 (2, 7)	6 (3, 10)	0.026
Hemoglobin (g/L)	90.0 (82, 102.5)	91.0 (77, 109.0)	0.499
Chronic comorbidities			
Diabetes	18 (18.0)	12 (18.8)	1.000
Hypertension	33 (33.0)	24 (37.5)	0.615
COPD	5 (5.0)	3 (4.7)	1.000
Coronary artery disease	16 (16.0)	7 (10.9)	0.490
CKD	6 (6.0)	7 (10.9)	0.374
Chronic liver disease	33 (33.0)	25 (39.1)	0.504
ACEI/ARB	3 (3.0)	6 (9.4)	0.156
Reason for ICU admission			
Surgical	62 (62.0)	50 (78.1)	0.030
Emergency	24 (24.0)	5 (7.8)	0.008
Medical	14 (14.0)	9 (14.1)	0.725
Mechanical ventilation	84 (84.0)	51 (79.7)	0.532
PaO_2_/FiO_2_	302.5 (192.0, 420.0)	288.3 (204.3, 413.7)	0.562
Sepsis	27 (27.0)	16 (25.0)	0.776
Vasopressors	22 (22.0)	10 (15.6)	0.420
Nephrotoxic drugs use	11 (11.0)	8 (12.5)	0.770
Baseline creatinine(µmol/L)	65.0 (53.9, 76.3)	65.4 (53.0, 76.0)	0.915
Serum creatinine diagnosing AKI (µmol/L)	113.0 (90.9, 144.0)	135.6 (105.1, 205.1)	0.001
eGFR^a^ (mL/min/1.73 m^2^)	58.6 ± 20.7	46.1 ± 22.9	0.001
UO 24 h after diagnosing AKI (mL/kg/h)	0.42 (0.36, 0.47)	0.27 (0.25, 0.36)	0.002
AKI classification			
Stage 1	73 (73.0)	18 (28.1)	< 0.001
Stage 2	22 (22.0)	32 (50.0)	
Stage 3	5 (5.0)	14 (21.9)	
Outcomes			
KRT in ICU	9 (9)	19 (30.6)	0.001
Hospital mortality	12 (12)	13 (20.3)	0.183
30-Day mortality	11 (11)	11 (17.2)	0.348

Values are median (Q1, Q3), mean ± SD or *n* (%). Nephrotoxic drug primarily includes vancomycin, aminoglycosides, rifampicin, amphotericin B, immunosuppressants and chemotherapy. ^a^Glomerular filtration rate was estimated by the Modification of Diet in Renal Disease formula

*APACHE II *Acute Physiology and Chronic Health Evaluation*, SOFA* Sequential Organ Failure Assessment, *COPD* chronic obstructive pulmonary disease, *CKD* chronic kidney disease, *ACEI* angiotensin-converting enzyme inhibitors, *ARB* angiotensin receptor blocker, *ICU* intensive care unit, *eGFR* estimated glomerular filtration rate, *UO* urine output, *AKI* acute kidney injury, *KRT* kidney replacement therapy

19 (30.6%) patients in renal non-recovery patients received KRT and 9 (9.0%) in renal recovery patients. Both hospital mortality and 30-day mortality were similar between the two subsets (12.0% vs. 20.3%, *p* = 0.183, and 11.0% vs. 17.2%, *p* = 0.348, respectively).

### Relationship between biomarker levels at enrollment and renal recovery

**Fig. 2:**
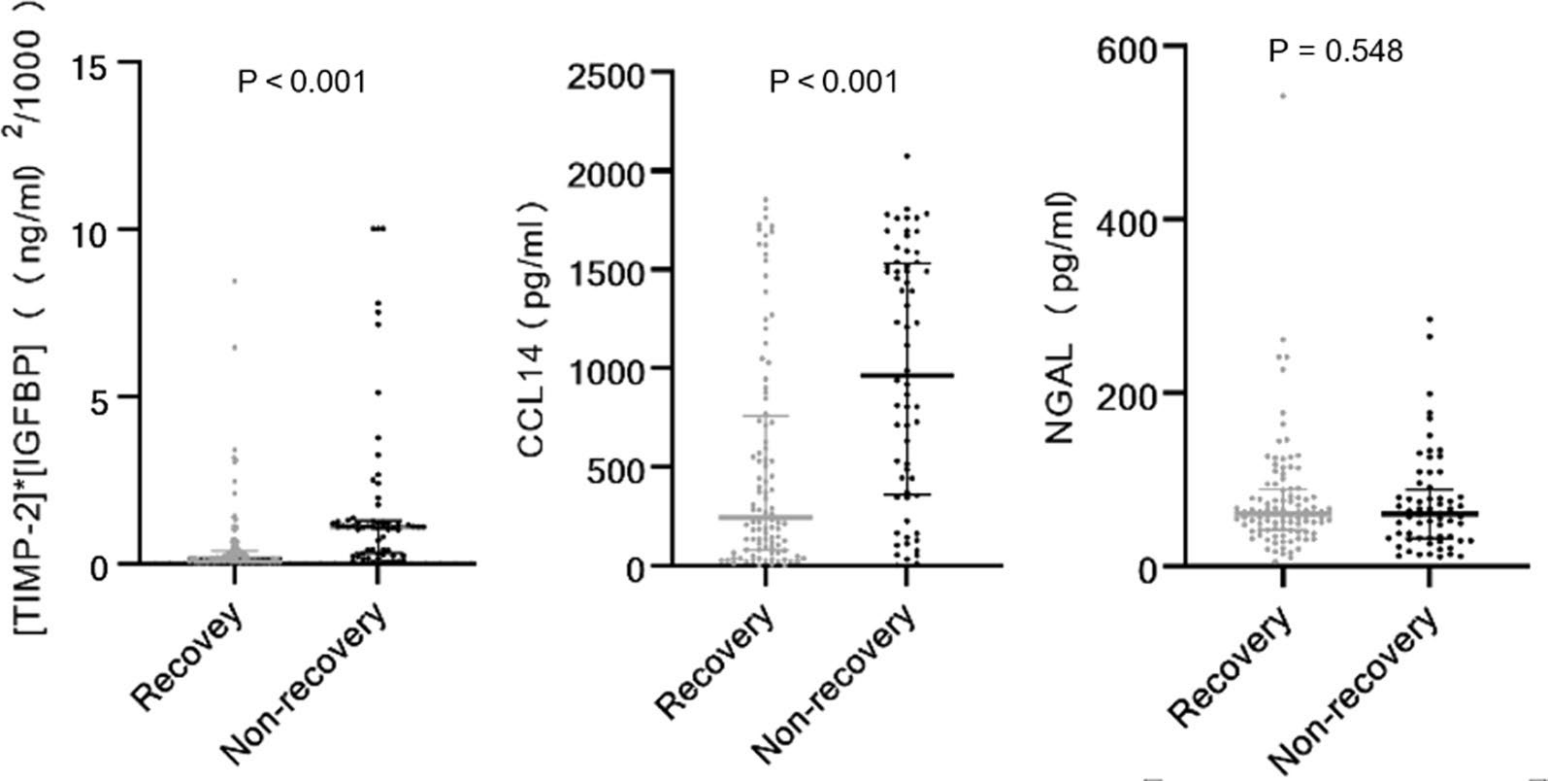
Urinary biomarkers levels on day 0 stratified by renal recovery. Day 0 means the day of AKI diagnosis*. TIMP-2* tissue inhibitor of metalloproteinases-2, *IGFBP-7* insulin-like growth factor-binding protein 7, CCL14 C–C motif chemokine ligand 14, NGAL neutrophil gelatinase-associated lipocalin

We compared urinary biomarker levels between the recovery and non-recovery groups (Fig. 2). Significant differences were observed in urinary biomarkers levels of [TIMP-2]*[IGFBP7] and CCL14. The patients with renal recovery showed levels of [TIMP-2] × [IGFBP7] and CCL14 were 0.12 (0.05, 0.37) [(ng/mL)^2^/1000] and 245.77 (80.15, 760.13) pg/mL, respectively. However, patients who failed to recover showed higher concentrations of [TIMP-2] × [IGFBP7] and CCL14 which were 1.09 (0.30, 1.28) [(ng/mL)^2^/1000] and 963.01 (359.91, 1531.04) pg/mL, respectively. Unfortunately, there was no significant difference in NGAL levels between the recovery group and non-recovery group (*p* = 0.548) and multivariate logistic regression analysis of biomarkers revealed that elevated urine NGAL was not an independent risk factor for renal recovery.

### Urinary biomarkers and prediction of renal recovery

[TIMP-2] × [IGFBP7] alone predicted renal non-recovery from AKI with an AUC of 0.78 (95% CI 0.71–0.84, *p* < 0.001). Its optimal threshold value was 0.72 [(ng/mL)^2^/1000] with a sensitivity of 65.6% and a specificity of 85.0% for predicting renal non-recovery. CCL14 alone predicted renal non-recovery from AKI with a lower AUC of 0.71 (95% CI 0.63–0.77, *p* < 0.001). Its optimal cutoff value was 625.69 pg/mL, with a sensitivity of 65.6% and a specificity of 72.0%. NGAL alone, however, had no predictive value for renal non-recovery with an AUC of 0.53 (95% CI 0.45–0.60, *p* = 0.562). For the multiple biomarkers model, combing CCL14 with [TIMP-2] × [IGFBP7] failed to help improve predictive performance, with a lower AUC than the [TIMP-2] × [IGFBP7] alone (Table 2).

**Table 2:** Predictive accuracy of urinary biomarkers on day 0 for renal non-recovery

Urinary Biomarkers	AUC	*p value*	Cutoff	Sensitivity (%)	Specificity (%)
	(95% CI)			(95% CI)	(95% CI)
[TIMP-2] × [IGFBP7]	0.78	< 0.001	0.72 (ng/mL)^2^/1000	65.6	85.0
	(0.71–0.84)			(52.7–77.1)	(76.5–91.4)
CCL14	0.71	< 0.001	625.69 (pg/mL)	65.6	72.0
	(0.63–0.77)			(52.7–77.1)	(62.1–80.5)
NGAL	0.53	0.562	37.69 (pg/mL)	34.4	81.0
	(0.45–0.60)			(22.9–47.3)	(71.9–88.2)
[TIMP-2] × [IGFBP7] + CCL14	0.77	< 0.001	0.224	90.6	51.0
	(0.70–0.83)			(80.7–96.5)	40.8–61.1

Day 0 means the day of AKI diagnosis*. AUC* area under the receiver operating characteristic, *CI* confidence interval, *TIMP-2* tissue inhibitor of metalloproteinases-2, *IGFBP-7* insulin-like growth factor-binding protein 7, *CCL14* C–C motif chemokine ligand 14, *NGAL* neutrophil gelatinase-associated lipocalin

According to previous studies, the widely used cutoff values for [TIMP-2] × [IGFBP7] to diagnose AKI were 0.3 ng/mL^2^/1000 for high sensitivity and 2.0 ng/mL^2^/1000 for high specificity [27, 28]. We tried to apply those cutoff values to predict renal non-recovery. When the cutoff was 2.0 ng/mL^2^/1000, no ability to predict renal non-recovery was observed. The sensitivity and negative predictive value (NPV) improved when the cutoff was modified to 0.3 ng/mL^2^/1000 (Additional file 1: Table S2).

We also explored whether urinary biomarkers could predict the incidence of secondary endpoints (Additional file 1: Table S3). Only urinary CCL14 predicted the initiation of KRT in ICU (AUC = 0.70, *p* = 0.001). None of the three biomarkers had the ability to predict 30 days and in-hospital mortality.

### Clinical risk prediction models for renal recovery

The univariate analyses showed that the APACHE II score, non-renal SOFA score, surgical and emergency reasons for ICU admission, serum creatinine, eGFR, urine output, and AKI stage might be risk factors for renal non-recovery (Table 1). There was a linear correlation between the APACHE II score and the non-renal SOFA score (*r* = 0.352, *p* < 0.001). According to a previous study, the non-renal SOFA score had better predictive value; therefore, the non-renal SOFA score was included in the multivariate logistic regression analysis [29]. Furthermore, urine output, eGFR, and serum creatinine levels were significantly associated with the AKI stage (*r* = 0.448, *p* = 0.002; *r* = − 0575, *p* < 0.001; and *r* = 0.6, *p* < 0.001, respectively) and determined AKI stage in clinical practice, so the AKI stage were included in multivariate analysis. The multivariate analyses showed the non-renal SOFA score and AKI stage were independent risk factors for renal non-recovery (Additional file 1: Table S4). We used any one of the factors or both two factors to construct clinical models for comparisons to find the best prediction model. The clinical risk prediction model joining the non-renal SOFA score with the AKI stage demonstrated the best AUC of 0.77 (95% CI 0.77–0.83, *p* = 0.004) for predicting renal non-recovery (Table 3).

**Table 3:** Stepwise analysis for prediction of non-recovery from AKI

Characteristic	Model	AUC (95% CI)	*p value*
Non-renal SOFA score	A	0.60 (0.52–0.68)	0.037
AKI Stage 2	B	0.74 (0.66–0.80)	< 0.001
Stage 3			
A + B		0.77 (0.70–0.83)	0.004

*CI* confidence interval, *AUC* area under the receiver operating characteristic, *SOFA* Sequential Organ Failure Assessment, *AKI* acute kidney injury

### Combining clinical and urinary biomarkers data

**Fig. 3:**
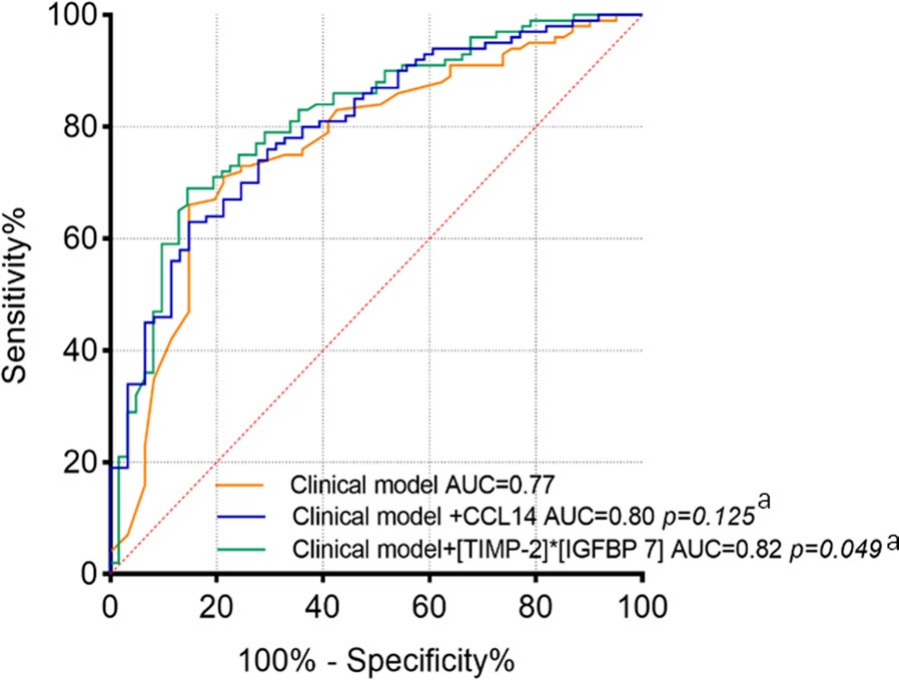
ROC curves of clinical model and corresponding urinary biomarkers model on day 0 for predicting renal non-recovery. Day 0 means the day of AKI diagnosis. ^a^Compared with clinical model. Clinical model included non-renal SOFA score and AKI stage. *AUC* area under the receiver operating characteristics curve, *CCL14* C–C motif chemokine ligand 14, *TIMP-2* tissue inhibitor of metalloproteinases-2, *IGFBP-7* insulin-like growth factor-binding protein 7

To determine the contributions of biomarkers when added to the clinical model, we compared the AUCs for models with and without biomarkers (Fig. 3). When [TIMP-2] × [IGFBP7] was combined with the clinical prediction model to predict renal non-recovery, the power was significantly improved, resulting in the best predictive AUC of 0.82 (95% CI 0.74–0.87, *p* = 0.049). When CCL14 was combined with the clinical prediction model, the power (AUC = 0.80, 95% CI 0.73–0.86, *p* = 0.125) failed to enhance.

We also assessed the capability of [TIMP-2]*[IGFBP7] and CCL14 to reclassify the degree of risk of recovery and non-recovery. Multivariate logistic regression analysis was used to calculate the probability of renal non-recovery based on models without and with biomarkers. Patients were stratified into three prespecified groups of “low,” “intermediate,” and “high” probability groups based on prediction models without biomarkers using cutoffs of < 30%, 30–60%, and > 60%, respectively. Then, we compared the proportions of reclassified patients across these three groups when biomarkers were introduced into models. For [TIMP-2] × [IGFBP7], 10.7% of patients were correctly reclassified into risk prediction categories by the biomarker-introduced model compared with the clinical model alone (*p* = 0.042). The IDI of [TIMP-2]*[IGFBP7] for renal non-recovery prediction was 8.0% (*p* = 0.003). However, the addition of CCL14 to the clinical model was unable to achieve significant improvement in predicting renal function reversal. Adding the two biomarkers at the same time to the clinical model also failed to improve renal non-recovery prediction (Table 4).

**Table 4:** NRI and IDI for assessing the contributions of different biomarkers for non-recovery prediction when combing with clinical model

Models	NRI (%)	*p value*	IDI (%)	*p value*
[TIMP-2] × [IGFBP7] + clinical model vs. clinical model	10.7	0.042	8.0	0.003
CCL14 + clinical model vs. clinical model	3.8	0.568	3.2	0.033
CCL14 + [TIMP-2] × [IGFBP7] + clinical model vs. clinical model	9.7	0.231	9.2	0.007

*NRI* net reclassification improvement, *IDI* integrated discrimination improvement, *TIMP-2* tissue inhibitor of metalloproteinases-2, *IGFBP-7* insulin-like growth factor-binding protein 7, *CCL14* C–C motif chemokine ligand 14

### Sensitivity analysis

Of 164 patients, 91 (55.5%) were diagnosed with AKI stage 1 and 73 (44.5%) were diagnosed with AKI at stages 2–3. We repeated the risk prediction analyses after removing patients with AKI stage 1. Urine [TIMP-2] × [IGFBP7] had a good predictive value in patients with AKI stages 2–3 and Urine CCL14 had a fair predictive value in patients with AKI stages 2–3 (Additional file 1: Table S5).

## Discussion

### Key findings

We performed a prospective study to explore the relationship between urinary biomarkers and renal non-recovery in critically ill patients with AKI and analyze the accuracy of a novel urinary biomarker (CCL14) and first-generation urinary biomarkers ([TIMP-2] × [IGFBP7] and NGAL) for predicting non-recovery. In this cohort, we found that both CCL14 and [TIMP-2] × [IGFBP7] levels were higher in patients who failed to recover from AKI. Higher CCL14 and [TIMP-2] × [IGFBP7] levels were independently associated with renal non-recovery. However, only the addition of [TIMP-2] × [IGFBP7] to the clinical model significantly improved predictive performance for renal non-recovery. Urine CCL14 failed to exceed [TIMP-2] × [IGFBP7] in the prediction of renal non-recovery. Urine NGAL may not be promising in predicting renal recovery in critically ill patients with AKI.

### Relationship to previous studies

To our knowledge, this is the first investigation of urine CCL14 in critically ill patients as a predictor of non-recovery from AKI. The previous study mainly concentrated on persistent AKI lasting more than 72 h, and the outcomes were also correlated with higher ill severity scores (non‑renal APACHE III score) and AKI stage at enrollment [12, 13]. Frustratingly, the AUC of CCL14 in the present study was lower than in previous studies. This may be related to the exclusion of patients with AKI stage 1 in the RUBY and SAPPHIRE studies. Functional AKI, meaning no damage or stress to the kidney, may occur more frequently in our setting and thus may explain the underperformance of the urine CCL14. Nevertheless, 28.1% of patients with AKI stage 1 in our study indeed experienced a renal non-recovery within 7 days, and our results provide a lesson for this population. Larger cohort studies are needed to validate the value of urine CCL14 in predicting renal recovery in AKI stage 1, or we can follow the example of Koyner et al. and find an appropriately raised standardized cut-off to achieve high specificity in identifying patients at high risk of renal non-recovery [30]. Moreover, our analysis essentially replicates the performance of urine [TIMP-2] × [IGFBP7] with the results of prior studies. When it was added to the clinical model consisting of non-renal SOFA score and AKI stage, the performance of predicting renal non-recovery was improved (AUC increased to 0.82) and further confirmed by NRI and IDI analysis. These results support the use of urine [TIMP-2]*[IGFBP7] for stratification of AKI patients in the ICU.

Currently, there is no definitive cutoff value for urinary CCL14 in the prediction of renal non-recovery. For urine CCL14, its cutoff value for predicting renal non-recovery is lower than previously reported values in the present study [14, 30]. The racial disparity may be linked to the threshold. For example, Stanley et al. observed variation in urine biomarkers of lupus nephritis across ethnicities [31]. All included patients in this study were Asian, which differed from European and American in the previous study. Another reason may be the selection of biomarker test kit. The NEPHROCLEAR CCL14 Test used in the previous study was different from the enzyme-linked immunosorbent assay kit used in this study. It is necessary for us to design a trial to explore racial disparity in urinary CCL14 using the same test kit in the future.

Urine NGAL has been proven to have good utility for predicting short-term and long-term kidney function reversal [32–34]. Yet, no significant difference in urine NGAL levels existed between the recovery and non-recovery groups in the present study (Fig. 2). We speculate that this is because NGAL is released by kidney epithelial cells and activated neutrophils during systemic inflammation [34], whereas the mentioned studies either included more than half of septic or infective patients or enrolled septic AKI participants. It is reasonable to measure inflammation-related AKI by inflammatory indicators, but in some studies similar to the present cohort, involving a majority of patients who underwent surgery before AKI, urine NGAL may be useless predictors for renal recovery from AKI [11, 35]. On the other hand, an earlier study has revealed that urine NGAL can be stored stably at − 80 °C for 6 months [36]. However, our study lasted more than one year, resulting in longer term storage of urinary samples. The instability of Urine NGAL during long-term storage may impact the discrimination of renal recovery [37].

### Implications of study findings

First, biomarkers are earlier than the elevated serum creatinine and oliguria, allowing a window of time when interventions might be able to prevent further injury. Second, our findings may have implications for design of intervention studies for AKI patients with high risk of renal non-recovery. Given the excellent performance of urine [TIMP-2] × [IGFBP7] in predicting AKI at high-risk populations, Zarbock et al. considered urine [TIMP-2] × [IGFBP7] ≥ 0.3 as inclusion criteria for intervention trial of the KDIGO bundle in a multicenter-center study enrolling 278 patients undergoing cardiac surgery and found a reduced occurrence of moderate and severe AKI in the intervention group compared with the control group [38]. Similarly, biomarker-directed intervention trials could be designed to confirm the benefit of the bundle interventions in patients with high risk of renal non-recovery. Third, our findings may have implications for clinical management of patients with AKI. Individuals with a high likelihood of recovery identified by biomarkers can have a regular dose of medication and no need for invasive monitoring. Therefore, early transfer out of the ICU is possible to reduce ICU-related complications, such as delirium, which was associated with greater mortality within 30 days of discharge [39].

### Strengths and limitations

Our study has several strengths. CKD is a risk factor for AKI, this study included not only new-onset AKI patients but also AKI patients with worsening preexisting CKD. Second, we tested multiple urinary biomarkers, which allowed us to visually compare a novel biomarker (CCL14) with first-generation markers. Third, the added value of [TIMP-2] × [IGFBP7] in renal non-recovery prediction was consistently found in multiple analyses, increasing our findings’ robustness.

Our study has, however, limitations. Although regression analysis showed two clinical parameters were independent risk factors for renal non-recovery in our study, the sample size was relatively small. We did not assess the predictive accuracy of urinary biomarkers in validation cohorts. Therefore, further studies will be needed to validate it. Moreover, this study included critically ill patients from two ICUs, which limits the generalizability of the findings. It was, however, performed in two tertiary hospitals, suggesting some degree of external validity for similar hospitals. In addition, we tested urinary biomarkers only on the day of AKI diagnosis, thus were unable to compare the kinetics of the three urinary biomarkers. We assessed the short-term prognosis but ignored the long-term prognosis, it would be better if we had explored the relationship between urinary biomarkers and the long-term prognosis of AKI.

## Conclusion

Urine CCL14 and [TIMP-2]*[IGFBP7] were fair predictors of renal non-recovery from AKI. Combing urine [TIMP-2] × [IGFBP7] with a clinical model of non-renal SOFA score and AKI stage enhanced the predictive performance for renal non-recovery. Urine CCL14 showed no significant advantage in predicting renal non-recovery compared to [TIMP-2] × [IGFBP7].

## Supplementary Information

**Additional file 1.** **Table S1.** Indications for initiation of KRT in patients with acute kidney injury. **Table S2.** Predictive accuracy of [TIMP-2]*[IGFBP7] for renal non-recovery at different cutoff values. **Table S3.** Predictions of secondary outcome of urinary biomarkers on day 0. **Table S4.** Multivariable regression analysis of clinical variables. **Table S5.** Urinary biomarkers on day 0 for predicting renal non-recovery in patients with AKI stage 2-3.

## Data Availability

All data generated or analyzed during this study are included in this published article and its additional information files.

## Abbreviations

AKI: Acute kidney injury
ICU: Intensive care unit
CCL14: C–C motif chemokine ligand 14
TIMP-2: Tissue inhibitor of metalloproteinase-2
IGFBP7: Insulin-like growth factor-binding protein 7
NGAL: Neutrophil gelatinase-associated lipocalin
AUC: Area under receiver operating characteristics curve
NRI: Net reclassification improvement
IDI: Integrated discrimination improvement
ROC: Receiver operating characteristic
NPV: Negative predictive value
CI: Confidence interval
CKD: Chronic kidney injury
GFR: Glomerular filtration rate
SCr: Serum creatine
KRT: Kidney replacement therapy
SOFA: Sequential organ failure assessment
