# Supplementary material for: Analysis of urinary C–C motif chemokine ligand 14 (CCL14) and first-generation urinary biomarkers for predicting renal recovery from acute kidney injury: a prospective exploratory study
Source: J Intensive Care. 2023 Mar 20;11:11. doi: 10.1186/s40560-023-00659-2 (PMC10026399; doi:10.1186/s40560-023-00659-2)
Supplement: Supplementary file 1 — Additional file 1. Table S1. Indications for initiation of KRT in patients with acute kidney injury. Table S2. Predictive accuracy of [TIMP-2]*[IGFBP7] for renal non-recovery at different cutoff values. Table S3. Predictions of secondary outcome of urinary biomarkers on day 0. Table S4. Multivariable regression analysis of clinical variables. Table S5. Urinary biomarkers on day 0 for predicting renal non-recovery in patients with AKI stage 2-3. [file 40560_2023_659_MOESM1_ESM.docx]

**Table S1** Indications for initiation of KRT in patients with acute kidney injury

| Volume overload | Acute severe pulmonary edema resistant to diuretic therapy |
| --- | --- |
| Metabolic acidosis | pH below 7.15 in a context of pure metabolic acidosis |
| Electrolyte abnormalities | Serum potassium concentration of more than 6.5 mmol/L |
| Uremia | Serum urea nitrogen of more than 112 mg/dl (40 mmol/L) |
| Persistent/progressive AKI | Oliguria or anuria for more than 72 hours |

*KRT* Kidney replacement therapy, *AKI* acute kidney injury

**Table S2** Predictive accuracy of [TIMP-2]*[IGFBP7] for renal non-recovery at different cutoff values

| **[TIMP-2]*[IGFBP7] cutoff (ng/ml^2^/1000)** | **AUC**  **(95% CI)** | **Sensitivity** | **Specificity** | **PPV** | **NPV** |
| --- | --- | --- | --- | --- | --- |
| 0.3 | 0.74 (0.66 - 0.80) | 76.6 (64.3 – 86.2) | 71.0 (61.1 – 79.6) | 62.8 (51.1 - 73.5) | 82.6 (72.9-89.9) |
| 0.72 | 0.78 (0.71 – 0.84) | 65.6 (52.7 – 77.1) | 85.0 (76.5 – 91.4) | 73.7 (60.3 – 84.5) | 79.4 (70.5 – 86.6) |
| 2.0 | 0.55 (0.47 – 0.63) | 18.8 (10.1 – 30.5) | 92.0 (84.8 – 96.5) | 60.0 (36.1-80.9) | 63.9 (55.5- 71.7) |

TIMP-2 tissue inhibitor of metalloproteinases-2, IGFBP-7 insulin-like growth factor-binding protein 7, *AUC* area under the receiver operating characteristic, *CI* confidence interval, *PPV* positive predictive value, *NPV* negative predictive value.

Table S3 Predictions of secondary outcome of urinary biomarkers on day 0

|  | **[TIMP-2]*[IGFBP7]** | | **CCL14** | | **NGAL** | |
| --- | --- | --- | --- | --- | --- | --- |
|  | **AUC (95% CI)** | **p-value** | **AUC (95% CI)** | **p-value** | **AUC (95% CI)** | **p-value** |
| Initiation of KRT | 0.59 (0.51 to 0.66) | 0.169 | 0.70 (0.62 to 0.77) | 0.001 | 0.59 (0.51 to 0.67) | 0.144 |
| 30-day mortality | 0.52 (0.44 to 0.60) | 0.738 | 0.63 (0.55 to 0.70) | 0.067 | 0.56 (0.48 to 0.64) | 0.416 |
| In-hospital mortality | 0.53 (0.45 to 0.61) | 0.659 | 0.57 (0.49 to 0.65) | 0.306 | 0.52 (0.44 to 0.60) | 0.783 |

TIMP-2 tissue inhibitor of metalloproteinases-2, IGFBP-7 insulin-like growth factor-binding protein 7, *CCL14* C-C motif chemokine ligand 14, *NGAL* neutrophil gelatinase-associated lipocalin, *AUC* area under the receiver operating characteristic, CI confidence interval, *KRT* kidney replacement therapy.

**Table S4** Multivariable regression analysis of clinical variables

| Variables | Odds Ratio (95% CI) | p-value |
| --- | --- | --- |
| Non-renal SOFA score | 1.08 | 0.048 |
| Reason for ICU admission |  |  |
| Medical | 1 [Reference] |  |
| Emergency | ns |  |
| Surgical | ns |  |
| AKI stage |  |  |
| Stage 1 | 1 [Reference] |  |
| Stage 2 | 5.92(2.61-13.41) | 0.001 |
| Stage 3 | 14.81(4.19-32.33) | 0.001 |

*SOFA*Sequential Organ Failure Assessment, *ICU* intensive care unit*, AKI* acute kidney injury, *CI* confidence interval, *ns* no significant.

**Table S5** Urinary biomarkers on day 0 for predicting renal non-recovery in patients with AKI stage 2-3

| **Biomarkers** | **AUC (95% CI)** | **Cut-off value** | ***p* value** |
| --- | --- | --- | --- |
| [TIMP-2]*[IGFBP7] ((ng/ml)^2^/1000) | 0.83 (0.72 - 0.91) | 0.38 | <0.001 |
| CCL14 (pg/ml) | 0.70 (0.58 - 0.80) | 594.39 | 0.002 |

Day 0 means the day of AKI diagnosis. *AKI* acute kidney injury, *AUC* area under the receiver operating characteristic, *CI* confidence interval, TIMP-2 tissue inhibitor of metalloproteinases-2, IGFBP-7 insulin-like growth factor-binding protein 7, *CCL14* C-C motif chemokine ligand 14.
